# A prospective single arm study of the effect of an acute oral glucose loading on the endothelial function of healthy participants

**DOI:** 10.1186/2251-6581-13-9

**Published:** 2014-01-07

**Authors:** Siew Yen Wong, Tayyab Hasan, Moi Ling Yong, Chee Fui Chong

**Affiliations:** 1 Institute of Health Sciences, Universiti Brunei Darussalam, Jalan Tungku Link, Gadong BE1410, Negara, Brunei Darussalam; 2 Department of Internal Medicine, Raja Isteri Pengiran Anak Saleha (RIPAS) Hospital, Jalan Tutong, Bandar Seri Begawan BA1710, Negara, Brunei Darussalam; 3 Department of Surgery, Raja Isteri Pengiran Anak Saleha (RIPAS) Hospital, Jalan Tutong, Bandar Seri Begawan BA1710, Brunei Darussalam

**Keywords:** Endothelial dysfunction, Diabetes, Atherosclerosis, Glucose load, Brachial artery flow mediated dilation

## Abstract

**Background:**

Hyperglycaemic load has been shown to cause endothelial dysfunction in patients diagnosed with diabetes mellitus or the pre-diabetic state of glucose intolerance. In the non-disease state such as in healthy subjects, the effect of glucose loading is still uncertain with conflicting results. The aim of this study was to test the hypothesis that an oral 75 g glucose load will not adversely attenuate the endothelial function of healthy participants, 2 hours postprandial.

**Methods:**

This is a prospective single arm study evaluating the brachial artery flow-mediated vasodilation of 12 healthy participants before and after a 75 g glucose loading. Participants’ age, body mass index, family history of diabetes, fasting blood glucose and 2 hour postprandial glucose levels were recorded. All data were analysed with SPSS 17.0 using Wilcoxon test.

**Results:**

Primary analysis of the participants’ brachial artery flow mediated vasodilation before and 2 hours after 75 g oral glucose loading did not show any statistically significant attenuation (*p* > 0.05) in brachial artery flow-mediated vasodilation, although a trend for reduction in endothelial relaxation was observed. Subgroup analysis of healthy participants with a positive family history of diabetes confirmed a statistically significant attenuation (*p* < 0.05) in brachial artery flow-mediated vasodilation after acute glucose loading even though the 2 hour postprandial blood glucose level, with a median value of 4.6 ± 2.2 mmol/L was within normal limits. This was not observed in the group without a positive family history of diabetes.

**Conclusion:**

Acute oral glucose loading significantly attenuates endothelial relaxation in healthy subjects with positive family history of diabetes but showed no effect in those without a positive family history of diabetes. The attenuation in endothelial relaxation was observed in the presence of normal glucose metabolism, suggesting a defect in endothelium relaxation even in the non-disease state in the group predisposed to diabetes.

## Background

Endothelial dysfunction is recognised as an early pathological process in the natural history of atherosclerosis, leading to long-term risk of cardiovascular events [1-3]. Postprandial hyperglycaemia, which is an independent risk factor for cardiovascular disease, is also accompanied by endothelial dysfunction [4,5]. It has been shown that due to chronic hyperglycaemia, there is endothelial dysfunction in individuals with insulin resistant, Type 2 diabetes mellitus [6-8], as well as in the pre-diabetic stage which includes both impaired fasting glucose and impaired glucose tolerance stage [8,9].

Endothelial dysfunction in these groups of individuals is already well established by the time they present for medical attention. Such endothelial dysfunction does not occur suddenly but is a process that gradually develops over time and may already be present even when the individual was healthy and disease free. The effect of glucose load on the endothelial function in healthy individuals has previously been studied but the available data have been conflicting. Some studies have suggested that a hyperglycaemic load attenuates endothelial function while more recent studies reported an absence of attenuation of endothelium function [9-16].

To better address with the pathological consequences of endothelial dysfunction, it is important that we understand what happens in the pre-disease state. Therefore, the purpose of this study was to test the hypothesis that transient acute glucose loading, induced by a 75 g oral glucose loading does not attenuate endothelial function in healthy individuals.

## Methods

This is a prospective single arm study assessing the effect of a 75 g oral glucose load on vascular endothelial function as measured by brachial artery flow-mediated vasodilation [BAFMD]), in healthy individuals recruited from the Raja Isteri Pengiran Anak Saleha (RIPAS) Hospital and from Universiti Brunei Darussalam (UBD). Twelve healthy participants between the ages of 18 and 30 were recruited for this study. Those with type 1 or type 2 diabetes mellitus or any known past medical history of cardiovascular diseases were excluded. Ethical approval to carry out this study was granted by the Medical and Health Research and Ethics Committee.

Participants were approached and briefed about the study and given a “Participant Information Sheet”. Upon agreement to participate, they were asked to sign the consent form provided. Demographic data such as age, gender, race, height, weight, medical history, and family history of diabetes mellitus, medications and any allergies were recorded. All data obtained during the BAFMD procedure were recorded in Microsoft Excel spreadsheet (Microsoft ®, USA) for data analysis.

Participants were instructed to fast from midnight and to withhold any agents that could affect vasoreactivity prior to the morning of the assessment [17]. These agents included analgesics, caffeine and non-steroidal anti-inflammatory medications [17]. Participants were also asked to rest well the night before, as it has been shown that stress has a negative effect on BAFMD [18,19].

For each participant, the BAFMD procedure was done twice. On the morning of the study, participants were taken to a quiet room with a constant ambient room temperature of 22°C and allowed to rest for 10–15 minutes prior to obtaining a blood specimen for fasting blood glucose level and then undergoing the baseline BAFMD. Following this, they were given an oral beverage containing 75 g of glucose (Trutol ®75 Glucose Tolerance Beverage, Thermo Scientific Inc. USA) and allowed to rest for two hours. After two hours, another blood specimen was taken for the 2 hour postprandial glucose level and the BAFMD procedure was then repeated. All blood specimens were analysed by the Biochemistry Laboratory at RIPAS hospital.

### Brachial artery flow mediated dilation (BAFMD)

This is a non-invasive technique used to assess endothelial function as described by Corretti et al. [17]. Participants were placed comfortably in a supine position with their left arm fully extended and cradled in a silicon-filled mould to prevent movement during the study. The ambient temperature of the room was kept at a constant of 22°C. The tourniquet pressure cuff (Torniquet 2500 ELC, VBM Medizintechnik,Germany) was placed 5-10 cm above the antecubital fossa of the left arm. A blood pressure cuff was placed on the right upper arm to monitor blood pressure before and after the procedure. An oxygen saturation probe was attached to the right thumb of the participant to monitor oxygen saturation and heart rate (Ultraview, Spacelabs Medical, U.S.A.).

The brachial artery was first palpated and then visualised using a B-mode ultrasound transducer (S-Nerve/Vascular^TM^ Ultrasound Scan, Sonosite Inc., U.S.A.). Once visualised, the position obtained by the transducer was marked. The best possible segment of the brachial artery was obtained and then the image was frozen at systole; indicated by maximum dilation of the artery. Using the caliper system in the ultrasound, the diameter of the brachial artery was measured. At least three readings were obtained along the artery and then the values averaged.

An initial baseline diameter of the brachial artery was measured (D_o_). Forearm ischaemia was achieved by inflating the tourniquet to a pressure of about 50-60 mmHg above the systolic blood pressure to occlude blood flow for 5 minutes. After 5 minutes, the cuff was deflated. The artery was again visualised and the image frozen at 1 minute (D_1_), 2 minutes (D_2_), 3 minutes (D_3_) and 5 minutes (D_5_) to measure the diameter at the respective times.

BAFMD was calculated as the percentage change of brachial artery diameter after cuff deflation relative to the baseline measurement:

BAFMD = [(D_X_-D_O_)/D_O_] × 100, where D_X_ is D_1_, D_2_, D_3_ and D_5_[17].

### Statistical analysis

The study sample size was calculated based on the alternative hypothesis that a 75 g glucose loading will significantly attenuate vascular endothelial vasodilatation. Based on this with a study power of 80% and an alpha value set at 0.05, to detect an expected difference in BAFMD of 2.8% (mean baseline BAFMD of 6.5% and a mean BAFMD of 3.7% two hours after a 75 g glucose loading), the sample size calculated was 5 [10]. However, in order to provide a sample size large enough to confirm our null hypothesis, a sample size of 12 was chosen.

All data collected were entered and analysed using SPSS 17.0 (SPSS Inc., California). The percentage change in BAFMD for each participant before and after the 75 g glucose load was analysed using the Wilcoxon test. A secondary subgroup analysis was carried out to compare BAFMD in participants with a family history of diabetes and in those without a family history of diabetes. Analysis was only carried out on 11 participants; one was excluded because the 2 hour blood glucose result could not be obtained due to difficulty in getting the blood sample (due to very fine veins). Statistical significance was set at a p value < 0.05.

## Results

### Sociodemographic characteristics

Table 1 summarizes the sociodemographic characteristics of all 11 participants. The participants’ ages ranged from 21 to 28 years, with a male to female ratio of 8:3. Participants did not have any known medical conditions and were not on any medications at the time of the study. Six participants had a positive family history of diabetes. Of these six participants, two of them had a first-degree relative with diabetes while the remainder had both first and second-degree relatives with diabetes. All participants had normal fasting and 2 hour postprandial blood glucose levels. [Normal fasting glucose: 3.5-6.0 mmol/L, 2 hour postprandial blood glucose < 7.8 mmol/L].

**Table 1:** Sociodemographic characteristics of participants according to negative family history of diabetes (n = 5) and those with positive family history of diabetes (n = 6)

**Variables**		**Negative FHx of diabetes**		**Positive FHx of diabetes**	
		**Median (IQR)**	**Frequency (%)**	**Median (IQR)**	**Frequency (%)**
**Age**		20.0 (2.0)^b^		21.5 (4.0)^b^	
**Gender**	Male		3 (60.0)		5 (83.3)
	Female		2 (40.0)		1 (16.7)
**Race**	Malay		4 (80.0)		1 (16.7)
	Chinese		0 (0.0)		3 (50.0)
	Others		1 (20.0)		2 (33.3)
**BMI (kg/m**^**2**^**)**		20.8 (3.9)		24.9 (8.7)	
**Oral glucose tolerance test**					
Fasting blood glucose		4.5 (0.8)		5.0 (0.4)	
2-hr postprandial blood glucose		4.4 (2.4)^a^		4.7 (1.8)	

^a^Skewed to the right.

^b^Skewed to the left.

FHx = Family history.

### Percentage change of BAFMD from baseline before and after glucose load

**Figure 1:**
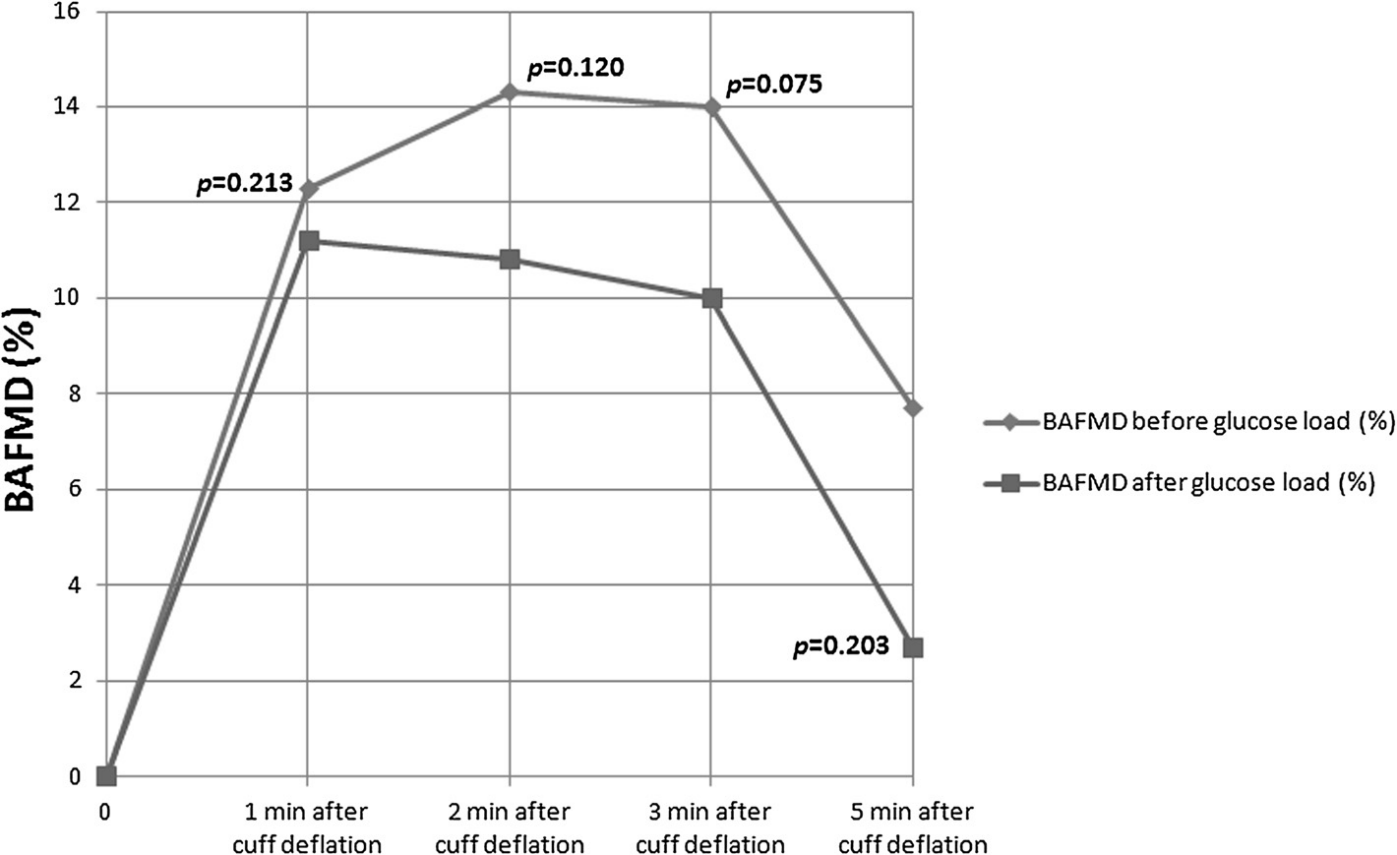
**BAFMD of participants before and after 75 g glucose load.** Legend: BAFMD of participants before and after 75 g glucose load (n = 11) showing a non-significant divergent trend at 2nd, 3rd and 5th minute of reperfusion.

Table 2 shows the overall analysis of BAFMD of the 11 participants. Although there was a trend towards reduced BAFMD 2 hour after glucose loading (maximum percentage change of BAFMD from baseline was 5% reduction), there was no statistical significance at all time intervals (*p* value at 1 minute, 2 minutes, 3 minutes and 5 minutes were all greater than 0.05; Figure 1). This confirms the hypothesis of the present study that endothelial function before and after the 75 g glucose load is not significantly attenuated.

**Table 2:** BAFMD values and its changes in participants before and after 75 g glucose load (n = 11)

**Variable**	**Median (IQR)**		***Z *** **Statistics**^**α**^	***p *** **value**^**α**^
	**Before 75 g glucose load**	**After 75 g glucose load**		
BAFMD, 1 min after cuff deflation (%)	12.3 (4.6)	11.2 (16.1)	−1.245	0.213
BAFMD, 2 min after cuff deflation (%)	14.3 (12.2)	10.8 (9.6)	−1.557	0.120
BAFMD, 3 min after cuff deflation (%)	14.0 (12.6)	10.0 (7.3)	−1.778	0.075
BAFMD, 5 min after cuff deflation (%)	7.7 (8.9)	2.7 (10.7)	−1.274	0.203

Baseline BAFMD (cm) – before oral glucose = 0.36 (0.13), after oral glucose = 0.35 (0.14).

^α^Wilcoxon test.

### Percentage change of BAFMD from baseline in participants with family history of diabetes

**Figure 2:**
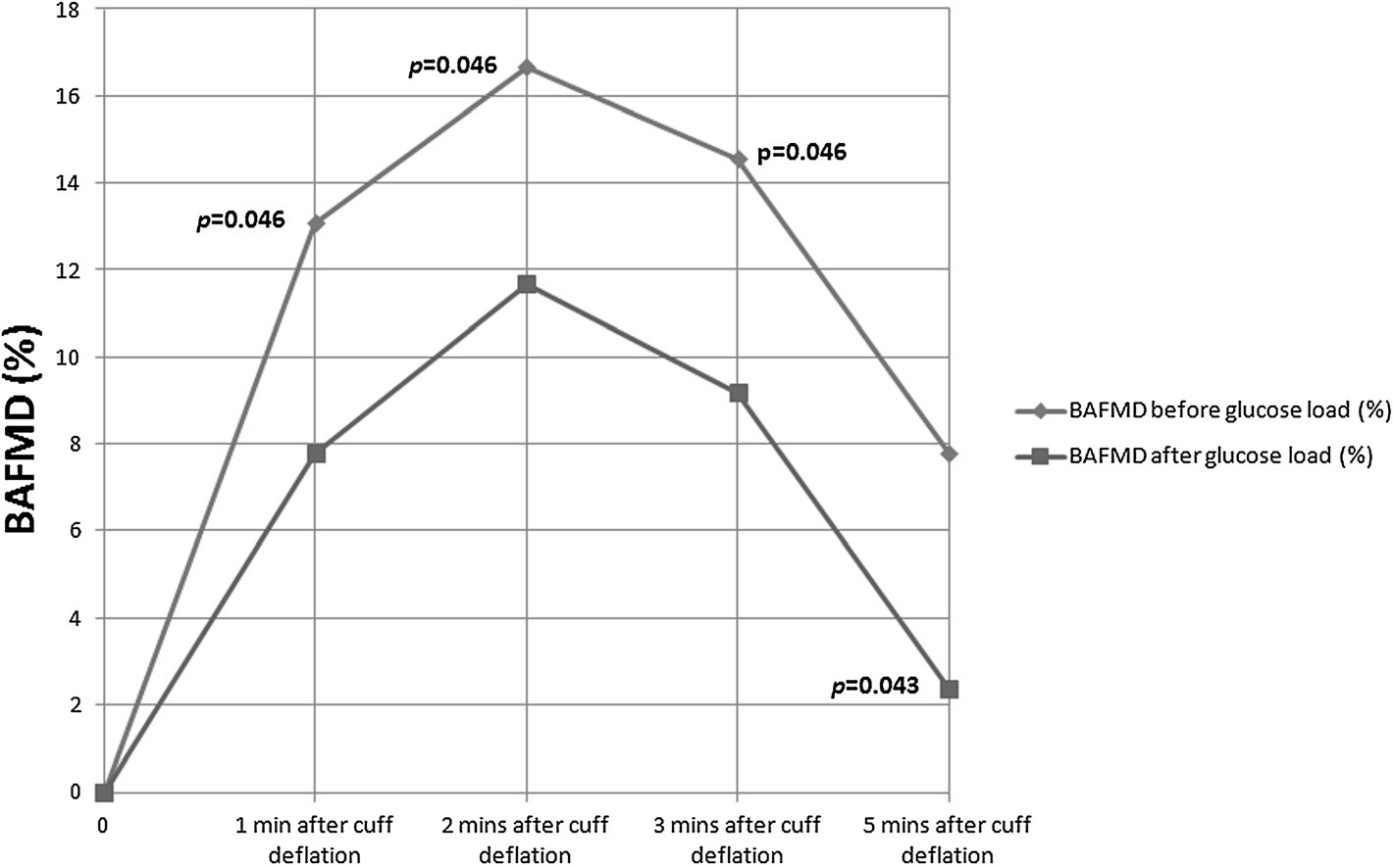
**BAFMD results of participants with family history of diabetes.** Legend: BAFMD results of participants with family history of diabetes (n = 6) showing significant reduction of BAFMD at all time intervals from 1 to 5 minutes of reperfusion 2 hour post prandial after oral glucose loading.

**Figure 3:**
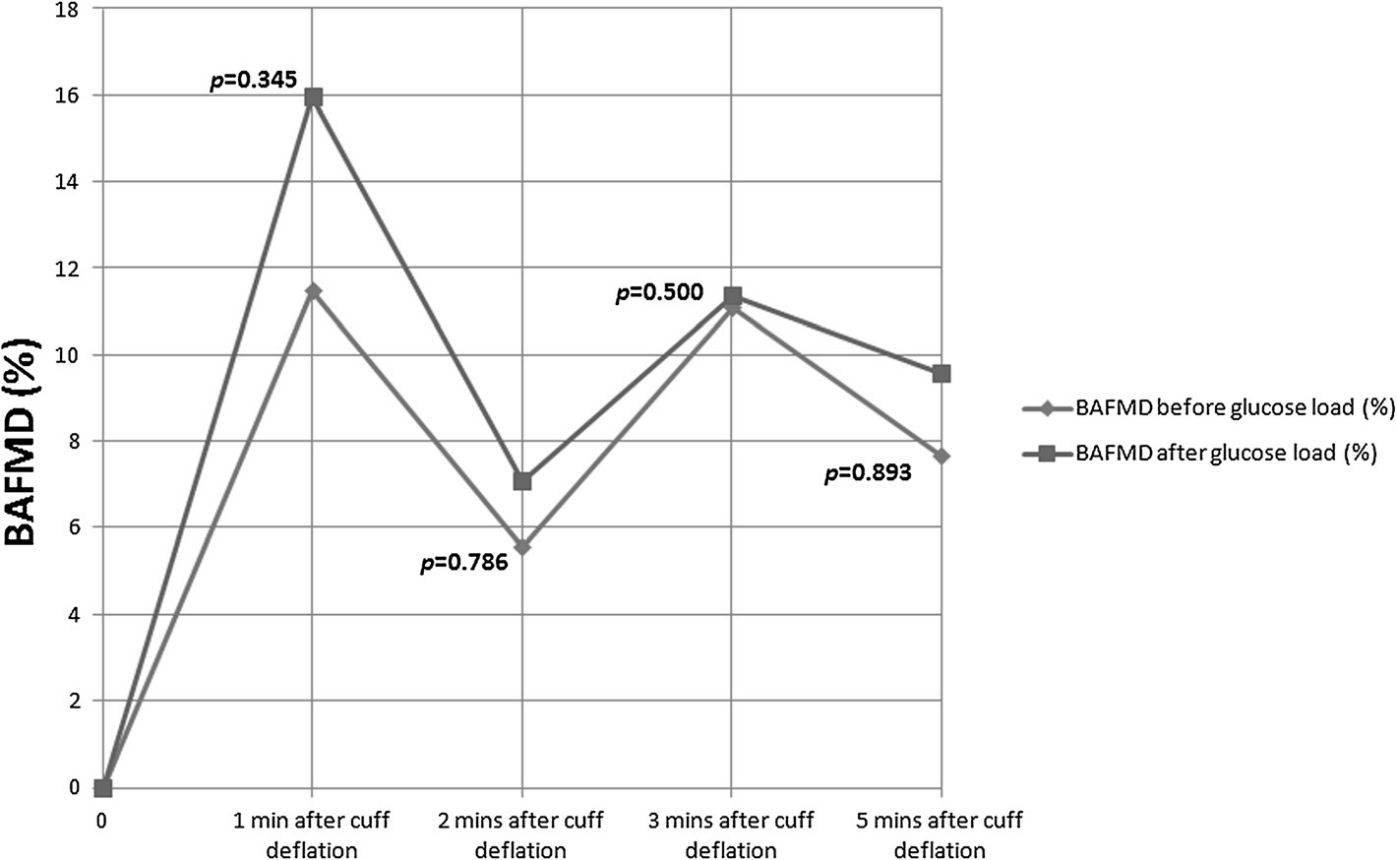
**BAFMD results of participants without family history of diabetes.** Legend: BAFMD results of participants without family history of diabetes (n = 5) showing no significant changes in BAFMD 2 hour post prandial after oral glucose loading.

Figures 2 and 3 summarize the recorded percentage change in BAFMD in participants with and without family history of diabetes respectively. Participants with a positive family history of diabetes showed significant reduction in the 2 hour postprandial BAFMD at all time intervals (*p* < 0.05 for 1 minute, 2 minutes, 3 minutes and 5 minutes; maximum percentage change of BAFMD from baseline was up 5.4% reduction; Figure 2). However, participants without a family history of diabetes did not show any statistically significant change in endothelial function after the glucose load (*p* > 0.05; maximum percentage change of BAFMD from baseline was up to 4.5% increase; Figure 3).

### Changes in heart rate and blood pressure before and after the 75 g glucose load

Table 3 shows no statistically significant changes in either heart rate (*p* = 0.8) or blood pressure (Systolic, *p* = 0.3; Diastolic, p = 0.7) during the study period.

**Table 3:** Changes in heart rate and blood pressure before and after the 75 g glucose load

**Variable**	**Median (IQR)**		***Z *** **Statistics**^**α**^	***p *** **value**^**α**^
	**Before 75 g glucose load**	**After 75 g glucose load**		
Heart rate	66 (10)	67 (12)	−0.178	0.859
Blood pressure - Systolic	113 (13)	113 (18)	−0.979	0.327
Blood pressure - Diastolic	72 (6)	73 (13)	−0.312	0.755

^α^Wilcoxon test.

## Discussion

The findings from our primary analysis support the hypothesis that endothelium-dependent vasodilation is not significantly affected by an oral glucose load in healthy individuals with normal glucose metabolism, concurring with more recent publications [15,16]. The difference between this study and earlier studies that do show attenuation in endothelial function after a glucose load may be due to differences in the method of glucose delivery [12,20,21].

In three studies, a forearm hyperglycaemic clamp method was used. Using this method, a dextrose solution was directly infused through a catheter into the brachial artery to be studied, in order to achieve a state of hyperglycemia [12,20,21]. The method used in all other studies involves giving an oral glucose load without mentioning whether a state of hyperglycemia was achieved [10,11,13-16]. The presence of an intravenous catheter may directly affect vascular endothelial function, hence may be a source of error.

Another potential issue is the different concentrations of glucose that were used in various studies. The optimum glucose concentration that would elicit a maximal change in FMD is not certain. Different concentrations may play an important role in determining endothelial function.

Hormonal variables, which could alter the physiology of the endothelium, were also controlled in some of these studies [16,21]. This included growth hormone, as well as pancreatic hormones such as glucagon and insulin. Both growth hormone and glucagon suppress the action of insulin and thus could influence endothelium dependent vasodilation. Reed et al., for example, administered somatostatin to their participants to suppress insulin and growth hormone [16]. Synthetic forms of these hormones were then replaced, thus ensuring constant levels throughout their study. In our study, we have not controlled these hormones but rather have allowed for normal physiological processes of glucose absorption and metabolism to occur.

Interestingly, our secondary subgroup analysis of the data from participants with a positive family history of diabetes showed a significant reduction in BAFMD at all time point, 2 hours post glucose loading, even though plasma glucose concentrations were within normal limits. This significant reduction may explain the divergent trend seen in the primary analysis in Figure 1. In the group without a positive family history of diabetes, baseline readings for BAFMD were almost parallel to those measured 2 hours after glucose loading, clearly indicating no effect of glucose loading in healthy subjects. None of the 11 participants recruited had any underlying medical conditions. They did not have any common risk factors associated with diabetes or cardiovascular diseases such as hypertension or hypercholesterolaemia, that could have accounted for the differences observed between the groups of participants with and without a family history of diabetes. It is noted that the group with positive family history of diabetes had a slightly higher BMI than the group without a positive family history of diabetes. This may suggest that the former group may have some predisposition to metabolic disorders, which could account for the significant changes seen. However, a separate subgroup analysis based on BMI >23 compared with those with BMI <23 did not showed any statistically significant changes in BAFMD. This did not support an assumption that higher BMI could have accounted for the significant changes in BAFMD seen in the group with a positive family history of diabetes and added further support to our assumption that it is the family history of diabetes that may be the determining factor. Changes in blood pressure and heart rate during the study can also account for changes in BAFMD but in our study group, there was no statistically significant change in both heart rate and blood pressure before and after the glucose load.

None of the earlier studies reporting attenuation of endothelial function with glucose loading or hyperglycemia in their healthy subjects have reported this association of family history of diabetes with endothelial dysfunction in response to glucose loading or hyperglycemia [10-14,20,21]. A study in 2006 by Goldfine et al. was the only other study to report similar attenuation of endothelial function in healthy subjects with positive family history of diabetes when exposed to glucose loading which lends further support to our assumption [21]. This failure to associate positive family history of diabetes with endothelial dysfunction in earlier studies (pre 2006) may also perhaps contribute to the conflicting results reported in literature. In the Goldfine study, endothelial dysfunction was reported in the presence of hyperglycaemia after glucose loading. This differs from our study in that, endothelial dysfunction occurred in the presence of normal post-prandial plasma glucose level, implying normal glucose metabolism.

Beckman et al. suggested that attenuation of endothelial function by hyperglycemia in normal healthy subject occurs in part, via protein kinase C-beta activation, which reduces endothelium derived nitric oxide synthesis [20]. Our findings of reduced endothelial relaxation in the presence of normal plasma glucose after acute glucose loading, may suggest that this pathway of protein kinase C-beta activation may already be present in healthy participants with a positive family history of diabetes even though they themselves have not developed signs and symptoms of abnormal glucose metabolism. Follow-up of these healthy participants with endothelial dysfunction should be done to see if they develop diabetes or even cardiovascular disease in the long-term. If confirmed, this study could help in identifying those people that may benefit from early lifestyle modifications including avoiding sudden, rapid glucose load like consuming carbonated drinks (soda) or sweets in excess, blood pressure and lipid control, weight management and smoking cessation to reduce their risk.

Because of the small sample size, we were unable to perform a subgroup analysis to compare the BAFMD in participants according to gender, age or race (Table 1). In addition this study was not designed to evaluate other factors that may contribute to endothelium-dependent flow mediated vasodilation such as hormones, dietary habits, environmental influences or exercise patterns. Future studies should consider a larger sample size for each of these factors to assess their significance.

## Conclusion

In healthy individuals with normal glucose metabolism and no family history of diabetes, oral glucose loading does not attenuate endothelial function. However, in healthy individuals with a family history of diabetes, despite normal glucose metabolism, endothelial function appears to be attenuated when exposed to an acute oral glucose load. Early intervention in this group of individuals may be beneficial in reducing their risk of developing diabetes and cardiovascular morbidity and mortality in the long-term.

## Abbreviations

BAFMD: Brachial artery flow-mediated dilation; DO: Diameter of brachial artery at time zero or baseline; D1: Diameter of brachial artery at 1 min after ischaemic reperfusion started; D2: Diameter of brachial artery at 2 min after ischaemic reperfusion started; D3: Diameter of brachial artery at 3 min after ischaemic reperfusion started; D5: Diameter of brachial artery at 5 min after ischaemic reperfusion started; Dx: Diameter of brachial artery at x min after ischaemic reperfusion started; FMD: Flow-Mediated Dilation; RIPAS: Raja Isteri Pengiran Anak Saleha; UBD: University Brunei Darussalam.

## Competing interests

The author(s) declare(s) that there is no competing interests.

## Authors’ contributions

Study investigator - WSY. Principal investigators and clinical supervisors – CFC and MLY. University supervisor - TH. All authors read and approved the final manuscript.

## Authors’ information

Co-authors – Tayyab Hasan and Moi Ling Yong.

Senior author – Chee Fui Chong.
